# Construction of Thiadiazole‐Linked Covalent Organic Frameworks via Facile Linkage Conversion with Superior Photocatalytic Properties

**DOI:** 10.1002/advs.202304697

**Published:** 2023-09-20

**Authors:** Shuailong Yang, Ziao Chen, Lei Zou, Rong Cao

**Affiliations:** ^1^ State Key Laboratory of Structural Chemistry Fujian Institute of Research on the Structure of Matter, Chinese Academy of Sciences Fuzhou Fujian 350002 China; ^2^ Fujian Science & Technology Innovation Laboratory for Optoelectronic Information of China Fuzhou Fujian 350108 China

**Keywords:** covalent organic frameworks, hydrogen evolution reaction (HER), linkage conversions, photocatalysis, thiadiazole

## Abstract

The establishment of facile synthetic routes to engineer covalent organic frameworks (COFs) with fully conjugated structure and excellent stability is highly desired for practical applications in optoelectronics and photocatalysis. Herein, a novel linkage conversion strategy is reported to prepare crystalline thiadiazole‐linked COFs via thionation, cyclization, and oxidation of *N*‐acylhydrazole bonds with Lawesson's reagent (LR). The as‐prepared thiadiazole‐linked COFs not only remain porosity and crystallinity, but enhance its chemical stability. Furthermore, thiadiazole‐linked COFs are more favorable to lower exciton binding energy and promote π‐electron delocalization over the whole reticular framework than *N*‐acylhydrazone‐linked COFs. Notably, the extended π‐conjugation structure and decent crystallinity of the resulting TDA‐COF are reflected by its higher photocatalytic H_2_ evolution rate (61.3 mmol g^−1^ in 5 h) in comparison with that (7.5 mmol g^−1^) of NAH‐COF.

## Graphical abstract

A facile and general strategy is developed to prepare thiadiazole‐linked covalent organic frameworks for photocatalytic H_2_ production. They serve as ideal platforms for understanding structure‐activity relationships toward the water‐splitting in the thiadiazole‐based polymer, which provide a new insight to rational design of COFs‐based photocatalyst for photocatalytic H_2_ production.

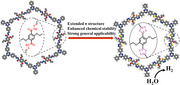

## 1 Introduction

Covalent organic frameworks (COFs) are a newly emerging class of crystalline porous materials synthesized by connecting various aromatic building blocks via covalent bonds.^[^ ^1^ ^]^ The precision in design and modification provided by reticular chemistry and versatile organic synthesis reaction has promoted the rapid development of COFs fields.^[^ ^2^, ^3^, ^4^ ^]^ A series of functional COFs have been constructed and widely applied in the field of gas adsorption, optoelectronics, catalysis, sensor, and so on.^[^ ^5^, ^6^, ^7^ ^]^ To develop advanced COF‐based materials in these fields, previous strategies mainly concentrate on the molecular design of the knot or linker with a specific linkage.^[^ ^8^, ^9^, ^10^, ^11^ ^]^ In addition to building blocks, however, linkage can also have a crucial effect on the stability, electronic structures, and morphologies of COFs materials.^[^ ^12^, ^13^, ^14^, ^15^, ^16^ ^]^ Over the past decades, there have been various covalent linkages used for the synthesis of COFs, such as benzoxazole,^[^ ^17^ ^]^ imidazole,^[^ ^18^ ^]^ dithiine,^[^ ^19^ ^]^ thiazole,^[^ ^20^ ^]^ indazole,^[^ ^21^ ^]^ and so on.^[^ ^22^, ^23^, ^24^ ^]^ However, high‐quality crystalline structures are usually achieved through the utilization of reversible covalent bonds in COFs.^[^ ^25^ ^]^ For instance, imine‐ and hydrazone‐linked COFs with partial π‐conjugation structure are reversibly formed by Schiff‐base reaction. Although the dynamic property of reversible covalent bonds offers a solution to the crystallization problem of COFs through error‐correction and self‐healing mechanism, the inherent reversibility and strong polarization of chemical bonds could result in bad chemical stability and blocked π‐electron delocalization throughout host networks.^[^ ^23^, ^24^, ^26^, ^27^ ^]^ To improve chemical stability and optimize photoelectronic properties of COFs, it is significant to expand the synthetic toolbox and construct COFs linked by irreversible and fully conjugated linkage.

**Scheme 1:**
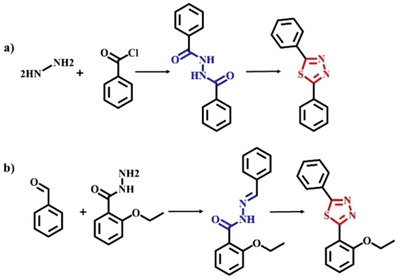
a) General preparation of diacylhydrazine and subsequent conversion into thiadiazole. b) Model reaction between 2‐ethoxybenzohydrazide and benzaldehyde to produce the *N*‐acylhydrazone‐based compound and final transformation into the thiadiazole derivative via the three‐step sequence of thionation, cyclization, and oxidation reaction.

Five‐membered 1,3,4‐thiadiazole (TDA) heterocycle, containing high electron affinity ability, great aromaticity, as well as excellent thermal and chemical stability, are extensively utilized in medicinal, organic electron, and optoelectronic chemistry.^[^ ^28^, ^29^ ^]^ Consequently, thiadiazole as a novel linkage in COFs will be encouraging by virtue of high chemical stability and superior electron transport ability. However, rapidly irreversible formation and cyclization of diacylhydrazines commonly result in amorphous thiadiazole‐based materials (**Scheme** **1**a), hence the crystalline thiadiazole‐based polymers remain largely unexplored.^[^ ^29^, ^30^, ^31^ ^]^ Furthermore, *N*‐acylhydrazone as a versatile linkage have obtained wide application in the field of photocatalysis, photoluminescence, and batteries, but these COFs still suffer from the inherent defect of the reversible bond.^[^ ^32^, ^33^, ^34^ ^]^ Meanwhile, the thionation of *N*‐acylhydrazone is a viable alternative method for the preparation of thiadiazole derivatives (Scheme 1b).^[^ ^35^ ^]^ Therefore, it will be a promising way to construct thiadiazole‐linked COFs through post‐synthetic modification of *N*‐acylhydrazone‐linked COFs.

As a proof‐of‐concept study, herein, we present a novel synthetic strategy for thiadiazole‐linked COFs from *N*‐acylhydrazone based‐COFs through thionation, cyclization, and oxidation of *N*‐acylhydrazone motifs using Lawesson's reagent (LR) as the thionating reagent. The thiadiazole was first introduced into COFs as a new linkage. Given the unique fully conjugated molecular structure and excellent chemical stability, the resulting COFs could demonstrate enormous potential as a semiconductor material for photocatalysis. Significantly, TDA‐COF displays excellent photocatalytic H_2_ evolution performance of 61.3 mmol g^−1^ in 5 h under visible‐light irradiation, which obviously outshines the 7.5 mmol g^−1^ of NAH‐COF under the same condition.

## 2 Results and Discussion

**Figure 1:**
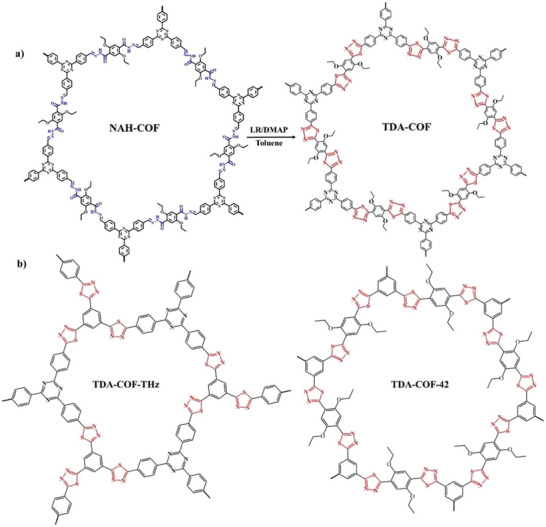
a) The scheme for the preparation of TDA‐COF via linkage conversion from *N*‐acylhydrazone to thiadiazole. b) Structure diagram of the other thiadiazole COFs synthesized via the method developed in this Work.

The preparation of TDA‐COF is performed by a two‐step synthesis process consisting of a reversible condensation reaction, followed by thionation, cyclization, and oxidative steps (**Figure**  **1**a). First, NAH‐COF is synthesized by the Schiff‐base reaction between 2,5‐diethoxyterephthalohydrazide and 1,3,5‐tris‐(4‐formyl‐phenyl)triazine in mesitylene/dioxane. Subsequent thionation, cyclization, and oxidation of the *N*‐acylhydrazone units in NAH‐COF into the fully conjugated thiadiazole linkage via LR in toluene afforded TDA‐COF. To showcase the broad potential of this method, two other *N*‐acylhydrazone linked COFs, NAH‐COF‐42 and NAH‐COF‐THz, were prepared and transformed to generate their respective thiadiazole‐linked COFs (Figure 1b; Figures S1–S4 and Tables S2 and S3, Supporting Information).

**Figure 2:**
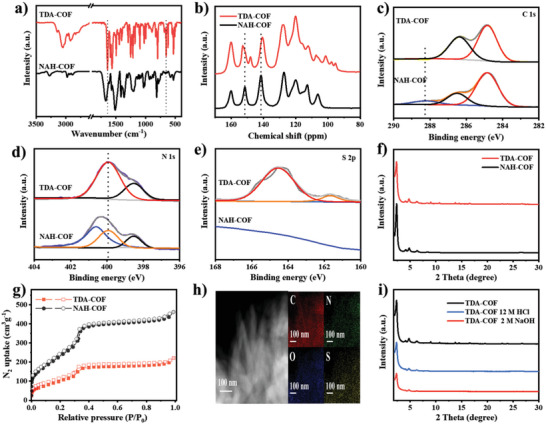
Structure characterization of NAH‐COF and TDA‐COF: a) FTIR spectra. b) Solid‐state ^13^C NMR spectra. c) C 1*s* XPS spectra. d) N 1*s* XPS spectra. e) S 2*p* XPS spectra. f) XRD patterns. g) The N_2_ sorption isotherms. h) HAADF‐STEM and corresponding EDX mappings of TDA‐COF. i) Chemical stability of TDA‐COF after treatment under strong acid and base conditions for 24 h.

The transformation of *N*‐acylhydrazone into the thiadiazole bonds was first investigated by Fourier‐transform infrared (FTIR) spectra. After the conversion of the *N*‐acylhydrazone units, two new peaks appeared at 1641 and 653 cm^−1^, which could be ascribed to the stretching vibration of C═N and C─S in thiadiazole linkage, respectively.^[^ ^36^, ^37^ ^]^ Correspondingly, the disappearance of C═O and C═N at 1670 and 1610 cm^−1^ in the *N*‐acylhydrazone motif, respectively, was observed, displaying that the *N*‐acylhydrazone units were converted into thiadiazole bonds (**Figure** **2**a).^[^ ^33^, ^34^, ^38^ ^]^ The solid‐state ^13^C NMR spectrum of NAH‐COF presented that C═O peak at 151.6 ppm and C═N peak at 141.6 ppm disappear, while TDA‐COF presented two new peaks at 152.7 and 148.1 ppm, which were ascribed to the thiadiazole carbon (Figure 2b).^[^ ^31^, ^35^ ^]^ In addition, the assignment of these peaks has been validated by comparison with the model compound (Figure S5, Supporting Information). Moreover, the X‐ray photoelectron spectroscopy (XPS) survey spectrum of NAH‐COF shows three different signals, corresponding to C, N, and O elements, while two extra signal of S element are discerned for TDA‐COF (Figure S6, Supporting Information). As for the high‐resolution C 1*s* XPS spectrum of NAH‐COF, the peak located at 288.2 eV corresponds to the carbon atom of C═O (Figure 2c).^[^ ^38^ ^]^ And it was obvious that C═O peak disappeared in TDA‐COF, which is in agreement with the FTIR spectrum. The N 1*s* XPS spectrum of NAH‐COF can be resolved into three peaks at 398.5, 399.9, and 400.6 eV, which can be assigned to triazine, imine, and amide N atom, respectively (Figure 2d).^[^ ^34^ ^]^ However, there are only two types of N atom for TDA‐COF, in which the peak (400.0 eV) corresponds to thiadiazole N atom. In addition, the deconvoluted S 2*p* XPS spectra of TDA‐COF present one binding energy peak at 164.6 eV, corresponding to thiadiazole S atom (Figure 2e).^[^ ^39^ ^]^ Therefore, the above evidences could well confirm the successful transformation of NAH‐COF into TDA‐COF.

The effect of post‐synthetic treatment on COF crystalline structures was studied via powder X‐ray diffraction (PXRD). TDA‐COF still retains decent crystallinity after the modification of NAH‐COF (Figure 2f). The experimental XRD of TDA‐COF was subjected to the Pawley refinements, yielding the unit cell parameters of *a* = *b* = 42.58 Å and *c* = 3.74 Å, with low *R*_wp_ = 6.28% and *R*_p_ = 4.33% (Figure S7a, Supporting Information). The simulated XRD patterns of TDA‐COF adopt the eclipsed AA stacking structure (Figure S7b, Supporting Information) and belong to hexagonal space group *P*3 (Table S1, Supporting Information). Furthermore, TDA‐COF features a porous hexagonal structure (Figure S7c, Supporting Information).

The permanent porosities of NAH‐COF and TDA‐COF were evaluated by N_2_ sorption isotherm at 77 K (Figure 2g). Both materials show an abrupt N_2_ uptake at very low relative pressure, indicating that they are microporous materials. The Brunauer–Emmett–Teller (BET) specific surface areas of NAH‐COF and TDA‐COF are as high as 690 and 350 m^2^ g^−1^, respectively. The decrease of BET could be attributed to the increase of the framework mass and reduction of pore volume and crystallinity.^[^ ^26^ ^]^ Furthermore, TDA‐COF pore sizes are marginally smaller than that of NAH‐COF (Figure S8, Supporting Information). Scanning/transmission electron microscopy (SEM/TEM) was conducted to visualize their structure, demonstrating that NAH‐COF and TDA‐COF hold similar rod morphology (Figures S9 and S10, Supporting Information). In addition, High‐angle annular dark field scanning transmission electron microscopy (HAADF‐STEM) imaging and energy‐dispersive X‐ray analysis (EDX) elemental mapping images substantiate uniform distribution of S element over TDA‐COF (Figure 2h), which is also indicative of the successful introduction of S element into COFs frameworks. Thermogravimetric analysis (TGA) profile evinces that NAH‐COF and TDA‐COF start to decompose at ≈380 °C (Figure S11, Supporting Information). However, after treatment with strong acid (12 m HCl) and base (2 m NaOH) solutions, the strong reflections in the PXRD patterns of NAH‐COF became disappeared (Figure S12, Supporting Information), showing the ruination of its crystal structures. In contrast, TDA‐COF retains crystallinity well, even after immersing in the same solvents for 24 h (Figure 2i). Apparently, the thiadiazole linkage can endow TDA‐COF with higher chemical stability.

**Figure 3:**
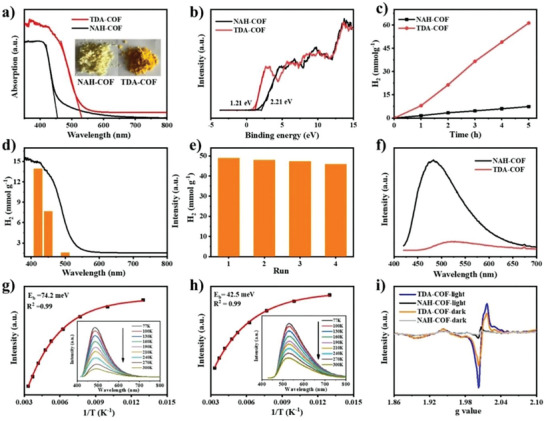
a) UV–vis DRS and optical images (the inset) of NAH‐COF and TDA‐COF. b) XPS valence spectra of NAH‐COF and TDA‐COF. c) Time‐dependent photocatalytic H_2_ performance of NAH‐COF and TDA‐COF (*λ* ≥ 420 nm). d) Wavelength‐dependent photocatalytic H_2_ production of TDA‐COF in 2 h. e) Cyclic stability of TDA‐COF with each run for 4 h. f) Steady‐state PL spectra of NAH‐COF and TDA‐COF. Temperature‐dependent PL spectra, *λ*_excitation_ = 400 nm of g) NAH‐COF and h) TDA‐COF (inset: corresponding fitting curves). i) EPR spectra of NAH‐COF and TDA‐COF in the dark and under visible‐light illumination.

To better understand the effect of the chemical conversions from *N*‐acylhydrazone bond to aromatic thiadiazole unit on the photoelectrical properties, we performed the UV–visible diffuse‐reflectance spectrum (UV–vis DRS) measurements for NAH‐COF and TDA‐COF. As shown in **Figure** **3**a, the absorption band edge of TDA‐COF is red‐shifted 71 nm relative to that of NAH‐COF, implying that thiadiazole linkage is more efficient in transmission of π‐conjugation over the frameworks than *N*‐acylhydrazone unit.^[^ ^23^ ^]^ The optical photo presents that TDA‐COF is golden yellow, while NAH‐COF is pale yellow. According to the Tauc plot, the optical band gap of TDA‐COF is 2.38 eV, which is significantly smaller than the 2.82 eV of NAH‐COF (Figure S13, Supporting Information). Furthermore, Mott–Schottky measurements were conducted to indicate that the flat band potentials of NAH‐COF and TDA‐COF are −0.56 and −0.89 V versus NHE, respectively (Figure S14a,b, Supporting Information). In addition, these results are consistent with that of valence band (VB) XPS (Figure 3b). The energy potential of TDA‐COF could thermodynamically meet the prerequisite of photocatalytic H_2_ generation and most other photocatalytic reactions (Figure S14c, Supporting Information). Meanwhile, the remarkable combination of pronounced π‐electron delocalization and excellent chemical stability makes TDA‐COF a promising candidate for visible‐light‐driven photocatalytic reactions. Furthermore, the effect of the thiadiazole units on the interaction with H_2_O molecular is explored by contact angle (Figure S15, Supporting Information), indicating that NAH‐COF and TDA‐COF have good hydrophilic character.^[^ ^40^, ^41^, ^42^ ^]^

Subsequently, to evaluate the photocatalytic activity of NAH‐COF and TDA‐COF, the photocatalytic H_2_ evolution reaction was performed using Au as a cocatalyst and triethanolamine (TEOA) as a sacrificial agent under visible‐light irradiation. The H_2_ evolution amount for TDA‐COF is 61.3 mmol g^−1^ in 5 h, while NAH‐COF presents an H_2_ production amount of 7.5 mmol g^−1^ under the identical conditions (Figure 3c). The H_2_ evolution rates for TDA‐COF are eight‐fold higher than that for NAH‐COF. The photocatalytic activity even surpasses most reported COF‐based photocatalytic systems (Table S4, Supporting Information). A similar phenomenon has also been observed in the TDA‐COF‐42 and TDA‐COF‐THz (Figure S16, Supporting Information), indicating that thiadiazole linkage is beneficial for photocatalytic H_2_ evolution. The wavelength‐dependent examination suggests that TDA‐COF has the potential to harvest a wide range of visible light. Specifically, the amount of H_2_ evolution under monochromatic light irradiation is 13.9, 7.7, and 1.6 mmol g^−1^ in 2 h at 420, 450, and 550 nm, respectively (Figure 3d). The apparent quantum yield (AQY) of TDA‐COF was evalutated to be 1.5% at 420 nm. Meanwhile, as shown in Figure 3e, no remarkable attenuation was observed after consecutive reaction lasting at least 16 h. Moreover, the FTIR spectra and XRD pattern of the recycled TDA‐COF are the same as those of the pristine sample (Figure S17, Supporting Information), indicating that the catalytic system is in possession of excellent robustness and outstanding durability in the photocatalytic H_2_ evolution reaction.

To explore the reason behind the superior performance of TDA‐COF over NAH‐COF, the steady‐state photoluminescence (PL) spectra were first examined (Figure 3f). When excited at 400 nm, the maximal emission peak of TDA‐COF is red‐shifted to 530 nm in comparison with NAH‐COF (481 nm) due to the decreasing of TDA‐COF band gap. Meanwhile, the PL intensity of TDA‐COF was much smaller than that of NAH‐COF, which demonstrates that charge recombination on TDA‐COF is severely suppressed. Furthermore, the average PL lifetime of TDA‐COF was decayed from 0.7 to 0.4 ns after linkage conversion (Figure S18, Supporting Information), promoting the transfer of photoexcited electron and suppressing radiative recombination of electron‐hole pair. To further illustrate the dynamics of charge recombination and separation, temperature‐dependent PL spectra were recorded. As shown in Figure 3g,h, the PL intensity augments with decreasing temperature.^[^ ^43^ ^]^ To evaluate the exciton binding energy (*E*_b_), the Arrhenius equation can be employed to fit the data, *I*(*T*) = *I*_0_/(1 + *A* exp(−*E*_b_/*k*_B_ *T*)). The *E*_b_ of TDA‐COF and NAH‐COF was estimated to be 42.5 and 74.2 meV, respectively. The lower *E*_b_ value suggests that non‐radiative pathways would occur more readily and less energy would be dissipated in the electron transfer process in TDA‐COF. Additionally, the generation of photoinduced charge carriers was characterized by electron paramagnetic resonance (EPR) spectroscopy.^[^ ^44^ ^]^ Specifically, the EPR signal intensity of TDA‐COF at *g* = 2.0083, corresponding to delocalized unpaired electron within the π‐conjugated COFs backbones, was stronger than that of NAH‐COF in the dark and under illumination (Figure 3i). Meanwhile, TDA‐COF demonstrates smaller semicircle radius (Figure S19a, Supporting Information) and higher photocurrent density (Figure S19b, Supporting Information) in comparison with NAH‐COF, indicating enhanced charge carrier transport and separation efficiency of TDA‐COF. In short, the above results show that the transformation of the reversible *N*‐acylhydrazone unit into the thiadiazole linkage could facilitate the π‐electron delocalization of COFs, which is favorable for visible light absorption, and lower exciton binding energy of COFs, which promotes exciton separation and transport. Thus, the TDA‐COF shows higher photocatalytic performance than the NAH‐COF counterpart.

## 3 Conclusion

In conclusion, we have developed a facile COF post‐synthetic modification strategy based on the thionation, cyclization, and oxidation reaction of *N*‐acylhydrazone to convert the reversible *N*‐acylhydrazone linkage into a fully conjugated and irreversible thiadiazole bond. In addition to retain the high crystallinity and ordered pore structure, the thiadiazole‐linked COFs demonstrate improved chemical stability, especially under strong acidic conditions. Importantly, the extended π‐electron delocalization by the formation of fully conjugated thiadiazole linkage facilitates visible‐light absorption, lower exciton binding energy, and suppresses recombination of the photogenerated electron‐hole pairs, leading to remarkably enhanced photocatalytic hydrogen evolution performance in comparison to its *N*‐acylhydrazone‐linked counterpart. This highly efficient approach allows for facile preparation of thadiazole‐linked COFs from widely available *N*‐acylhydrazole‐linked COFs, laying the foundation for investigating practical applications of thiadiazole‐linked COFs material.

## 4 Experimental Section

Experimental details are shown in the Supporting Information.

## Conflict of Interest

The authors declare no conflict of interest.

## Supporting information

Supporting Information

## Data Availability

The data that support the findings of this study are available from the corresponding author upon reasonable request.
