# Supplementary material for: Construction of Thiadiazole‐Linked Covalent Organic Frameworks via Facile Linkage Conversion with Superior Photocatalytic Properties
Source: Adv Sci (Weinh). 2023 Sep 20;10(31):2304697. doi: 10.1002/advs.202304697 (PMC10625113; doi:10.1002/advs.202304697)
Supplement: Supplementary file 1 — Supporting Information [file ADVS-10-2304697-s001.pdf]

ADVANCED SCIENCE
Open Access

Supporting Information

for *Adv. Sci.*, DOI 10.1002/advs.202304697

Construction of Thiadiazole-Linked Covalent Organic Frameworks via Facile Linkage Conversion with Superior Photocatalytic Properties

*Shuailong Yang, Ziao Chen, Lei Zou and Rong Cao**

Copyright WILEY-VCH Verlag GmbH & Co. KGaA, 69469 Weinheim, Germany, 2023.

Construction of Thiadiazole-Linked Covalent Organic Frameworks via Facile Linkage Conversion with Superior Photocatalytic Properties

Shuailong Yang, Ziao Chen, Lei Zou, Rong Cao*

Experimental section

General Information. All chemicals were commercially available and used without further purification. The 2,5-diethoxyterephthalohydrazide, Lawesson's reagent and H_2AuCl_6 were purchased from Adamas (China). 4-Dimethylaminopyridine was purchased from Shanghai Macklin Biocheemical Co., Ltd. 1,3,5-tris-(4-formyl-phenyl)triazine and benzene-1,3,5-tricarbaldehyde were purchased from Shanghai Kaiyulin Pharmaceutical Technology Co., Ltd. Benzene-1,3,5-tricarbohydrazide was synthesized according to reported literature.[1] Solid-state ^{13}C CP/MAS NMR was performed on a Bruker SB Avance III 500 MHz spectrometer with a 4-mm double-resonance MAS probe. Fourier transform infrared (FTIR) spectra were recorded with KBr pellets using Perkin-Elmer Instrument. Solid-state UV-Vis diffuse reflectance spectra (UV-Vis DRS) were recorded at room temperature using UV-Vis Varian Cary 500 spectrophotometer. Barium sulfate was used as a reference sample. The steady-state photoluminescence (PL) spectra were measured by an Edinburgh FL/FS1000 spectrophotometer and time-resolved PL spectra were recorded upon 375 nm excitation. Thermogravimetric analysis (TGA) measurement was carried out on SDT Q600 by heating samples from 30 to 800 ^{o}C in dynamic nitrogen atmosphere with heating rate of 10 °C·min^{-1}. Nitrogen adsorption and desorption isotherms were measured at 77 K using a Micromeritics ASAP 2020 system. The samples were degassed at 120 ^{o}C for 10 h before the measurements. Specific surface areas were calculated from the adsorption data using Brunauer-Emmett-Teller (BET) equation. The calculation of the pore size distribution was done using the quenched-solid density functional theory (QSDFT) equilibrium model. Field-emission scanning electron microscopy (SEM) and transmission electron microscopy (TEM) were performed on a JEOL JSM-7500F operated at an accelerating voltage of 3.0 kV. X-ray photoelectron spectroscopy (XPS) measurements were performed on Thermo ESCALAB 250 spectrometer using non-monochromatic Al Kα X-rays as the excitation source and choosing C 1s (284.8 eV) as the reference line. The photocatalytic H_2 production was irradiated under high precision xenon

illuminator system (LX300F) with λ ≥ 420 nm cutoff filter unless otherwise stated. The PL-MW-2000 spectroradiometer (Beijing Perfectlight) was used to measure the intensity of incident monochromatic illumination. Gas chromatographic analysis was conducted using Aglient (7820A) equipped with flame ionization detector (FID) and thermal conductivity detector (TCD) column.

Structure simulation of TDA-COF, TDA-COF-42 and TDA-COF-THz. The structure models of TDA-COF, TDA-COF-42 and TDA-COF-THz were generated using Materials Studio programs. The initial unit cell dimension was set to the theoretical parameters. The Le Bail refinement was performed to optimize lattice parameters iteratively until the Rwp value converges and the overlay of the refined profiles showed good agreement. The atomic positions and total energies were fully optimized using Forcite module of Materials Studio. The final crystal structure was then optimized using the Castep module of Materials Studio.

Synthesis of (E)-benzylidene-2-ethoxybenzohydrazide. The compound was synthesized according to the literature methods.[S1] Benzaldehyde (154 μL, 1.5 mmol) and 2-ethoxybenzohydrazide (270 mg, 0.75 mmol) were dissolved in chloroform (50 mL). After trifluoroacetic acid (30 μL) was added, the mixture was heated to reflux for 4 h and then cooled to room temperature. Hexane were added to the solution and then white solid are precipitated. The solid was filtered, washed with diethyl ether to generate the target product. ^{1}H NMR (400 MHz, $CDCl_3$): δ 11.12 (s, 1H), 8.32 (d, 1H), 8.13 (s, 1H), 7.80 (m, 2H), 7.47 (m, 1H), 7.40 (m, 3H), 7.12 (t, 1H), 6.98 (d, 1H), 4.28 (q, 2H) and 1.62(t, 3H).

Synthesis of 2-(2-ethoxyphenylene)-5-phenyl-1,3,4-thiadiazole. The compound was synthesized according to the literature methods.[S2] To the (E)-benzylidene-2-ethoxybenzohydrazide (134 mg, 0.5 mmol), Lawesson's reagent (162 mg, 0.4 mmol) and toluene (10 mL) were added, followed by 4-(dimethylamino)pyridine (74mg, 0.6 mmol). The resulting mixture was refluxed for 24h. After evaporation of toluene, the residue was purified by flash-column chromatography. ^{1}H NMR (400 MHz, $CDCl_3$): δ 8.55 (q, 1H), 8.03 (m, 2H),

7.45 (m, 4H), 7.11 (t, 1H), 7.02 (d, 1H), 4.27 (q, 2H) and 1.61(t, 3H).

The synthesis of NAH-COF. The NAH-COF was synthesized according to the literature methods.[S3] A Pyrex tube was charged with 1,3,5-tris-(4-formyl-phenyl)triazine (17.7 mg, 44 μmol), 2,5-diethoxyterephthalohydrazide (18.6 mg, 66 μmol), 330 μL of anhydrous dioxane and 660 μL of mesitylene. The tube was immersed in an ultrasonic bath for 15 min; following sonication 100 mL of 6 M aqueous acetic acid were added. The tube was flash frozen at 77 K (liquid N_2 bath), degassed under freeze-pump-thaw for three cycles. The tube was flame sealed and heated at 120 °C for three days. These solid at the bottom of the tube which was isolated either by filtration and washed with anhydrous THF and anhydrous acetone.

The synthesis of NAH-COF-42. The NAH-COF-42 was synthesized according to the literature methods.[S4] A Pyrex tube was charged with benzene-1,3,5-tricarbaldehyde (5 mg, 44 μmol), 2,5-diethoxyterephthalohydrazide (18 mg, 65 μmol), 250 μL of anhydrous dioxane and 750 μL of mesitylene. The tube was immersed in an ultrasonic bath for 15 min; following sonication 100 mL of 6 M aqueous acetic acid were added. The tube was flash frozen at 77 K (liquid N_2 bath), degassed under freeze-pump-thaw for three cycles. The tube was flame sealed and heated at 120 °C for three days, yielding a pale-yellow solid at the bottom of the tube which was isolated either by filtration and washed with anhydrous dioxane and anhydrous acetone.

The synthesis of NAH-COF-THz. The NAH-COF-THz was synthesized according to the literature methods.[S1] A Pyrex tube was charged with 1,3,5-tris-(4-formyl-phenyl)triazine (19.8 mg, 50 μmol), benzene-1,3,5-tricarbohydrazide (12.6 mg, 50 μmol), 1 mL of anhydrous dioxane and 1mL of mesitylene. The tube was immersed in an ultrasonic bath for 15 min; following sonication 0.2 mL of 6 M aqueous acetic acid were added. The tube was flash frozen at 77 K (liquid N_2 bath), degassed under freeze-pump-thaw for three cycles. The tube was flame sealed and heated at 120 °C for three days. These solid at the bottom of the tube which was isolated either by filtration and washed with anhydrous THF and anhydrous

acetone.

The synthesis of TDA-COF. The TDA-COF was synthesized according to the literature methods.[S2] To the NAH-COF (39 mg, 0.5 mmol), Lawesson's reagent (100 mg) and toluene (20 mL) were added, followed by 4-(dimethylamino)pyridine (44 mg). The resulting mixture was refluxed for 24 h. The resultant solid was isolated by filtration and washed with DMF and THF and dried under vacuum at 70 ^{o}C. Next, the solid was re-dispersed into 20 mL toluene, followed by adding the same amount of Lawesson's reagent and 4-(dimethylamino)pyridine and was refluxed for 24 h. And the resultant solid was isolated by filtration and washed with DMF and THF and dried under vacuum at 70 ^{o}C.

The synthesis of TDA-COF-42. The TDA-COF-42 was synthesized according to the literature methods.[S2] To the NAH-COF-42 (30 mg), Lawesson's reagent (100 mg) and toluene (20 ml) were added, followed by 4-(dimethylamino)pyridine (44 mg). The resulting mixture was refluxed for 24 h. The resultant solid was isolated by filtration and washed with DMF and THF and dried under vacuum at 70 ^{o}C. The above step are again repeated twice and total reaction time are 72 h.

The synthesis of TDA-COF-THz. The TDA-COF-42 was synthesized according to the literature methods.[S2] To the NAH-COF (34 mg), Lawesson's reagent (100 mg) and toluene (20 mL) were added, followed by 4-(dimethylamino)pyridine (44 mg). The resulting mixture was refluxed for 24 h. The resultant solid was isolated by filtration and washed with DMF and THF and dried under vacuum at 70 ^{o}C. The above step are again repeated twice and total reaction time are 72 h.

Photoelectrochemical measurement. To fabricate the working electrode, 2 mg of fully ground sample and 2 mL of ethanol (5 % Nafion) were mixed under sonication for 30 min to make the sample fully dispersible. The resultant slurry was dropped onto a piece of fluoride-tin oxide (FTO) glass substrates with cover area of 1 cm^2. The uncovered parts of the electrode were coated with epoxy. The working electrode was dried in air naturally. The

photocurrents were recorded by an electrochemical workstation (CHI650E) equipped with a conventional three-electrode cell. A platinum plate electrode and an Ag/AgCl electrode were used as the counter electrode and the reference electrode, respectively. The electrodes were immersed in 0.2 M aqueous Na_2SO_4 solution. The working electrode was illuminated by high precision xenon illuminator system (LX300F) with a 420 nm cut-off filter from the backside to minimize the impact of thickness of the semiconductor layer. Each measurement was repeated three times under ambient conditions.

Photocatalytic measurement. TDA-COF (5 mg) was dispersed in the solution of H_2O and triethanolamine (5 mL, 4:1) in a quartz reactor. $HAuCl_4$ (10 µL, 9 wt% in H_2O) as precursor for in situ formation of Au cocatalyst was added. The quartz reactor was degassed with vacuum pump and then backfilled with Ar. This process was repeated three times. After the last cycle, the flask was backfilled with Ar (1.0 atm). The temperature of the reaction solution was maintained at 25 ^{o}C and the reaction mixture was irradiated under Xe lamp with $\lambda \geq 420$ nm cutoff filter. After reaction, the gases were analyzed by Aglient (7820A). Cycling photocatalytic tests were performed using the same way after the samples were collected and washed with water.

Determination of apparent quantum efficiency. The apparent quantum efficiency (AQE) of the reaction system was measured using a Xe lamp with 420 nm band-pass filter (high precision xenon illuminator system (LX300F)) and the PL-MW-2000 spectroradiometer (Beijing Perfectlight) was used to measure the intensity of incident monochromatic illumination (30 mW cm^{-2}). The irradiation area are 3.14* 4 cm^2. Depending on the amount of CO evolution per hour, AQE was calculated as follow:

$$AQE\ \% = 2 \times (n_e \cdot N_A \cdot h \cdot c) / (I \cdot S \cdot t \cdot \lambda) \times 100\%$$

Where, n_e is the amount of generated electrons for H_2, N_A is Avogadro constant (6.02×10^{23} mol^{-1}), h is the Planck constant (6.626×10^{-34} J·s), c is the speed of light (3×10^8 m s-1), S is the irradiation area (cm^2), I is the intensity of irradiation light (W cm^{-2}), t is the photoreaction

time (s), λ is the wavelength of the monochromatic light (420 nm)."

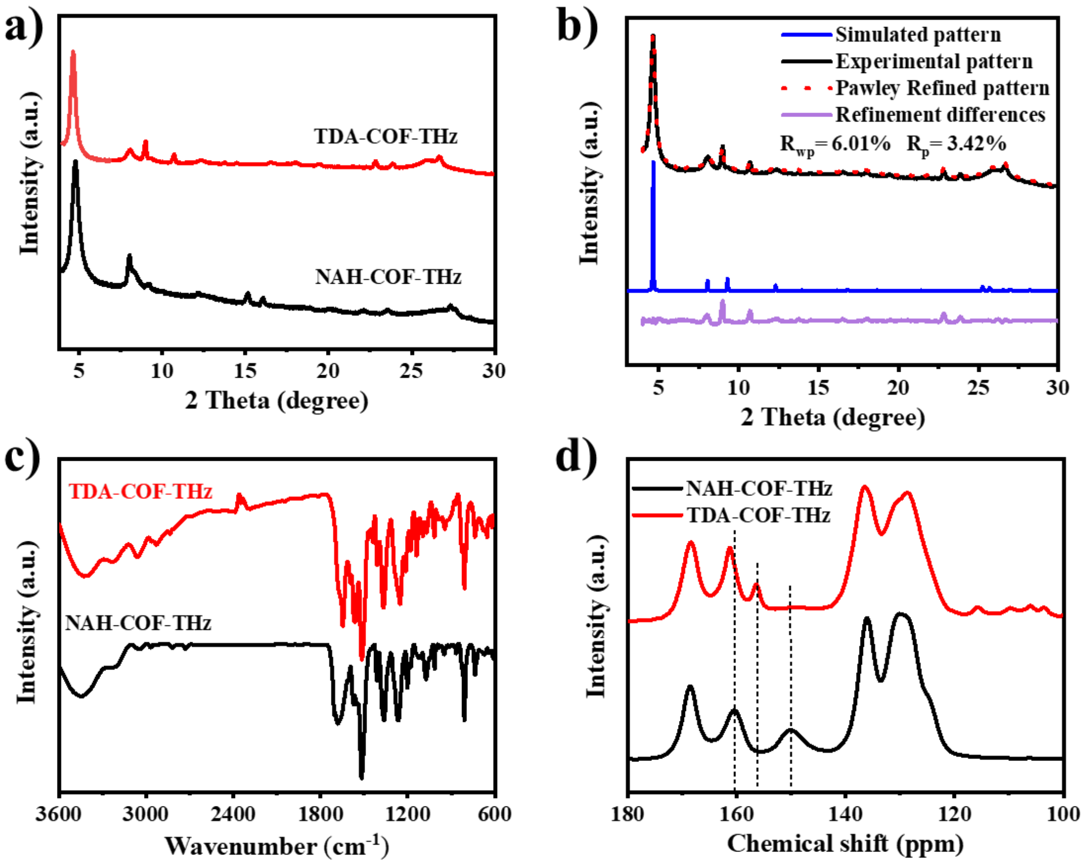

Figure S1. Characterization of NAH-COF-THz and TDA-COF-THz: a). XRD patterns. b) Experimental and simulated powder X-ray diffraction patterns of TDA-COF-THz. c) FTIR spectra. d) Solid-state ^{13}C NMR spectra.

TDA-COF-THz can maintains good crystallinity after the modification of NAH-COF-THz (Figure S1a). Furthermore, the Pawley refinements of TDA-COF-THz give the unit cell parameters of a = b = 21.96 Å and c = 3.52 Å, Rwp = 6.01% and Rp = 3.42% (Figure S1b and Table S2). In the Fourier-transform infrared (FTIR) spectra (Figure S1c), two new peak appeared at 1649 and 654 cm^{-1}, which could be ascribed to the stretching vibration of C=N and C-S in thiadiazole linkage, respectively. Correspondingly, disappearance of C=O and C=N at 1681 and 1610 cm^{-1} in *N*-acylhydrazone motif. In the solid-state ^{13}C NMR spectrum, C=O peak at 160.3 ppm and C=N peak at 150.1 ppm disappear for NAH-COF-THz, while TDA-COF-THz presented two new peaks at 161.3 and 156.2 ppm, which were ascribed to the thiadiazole carbon (Figure S1d).

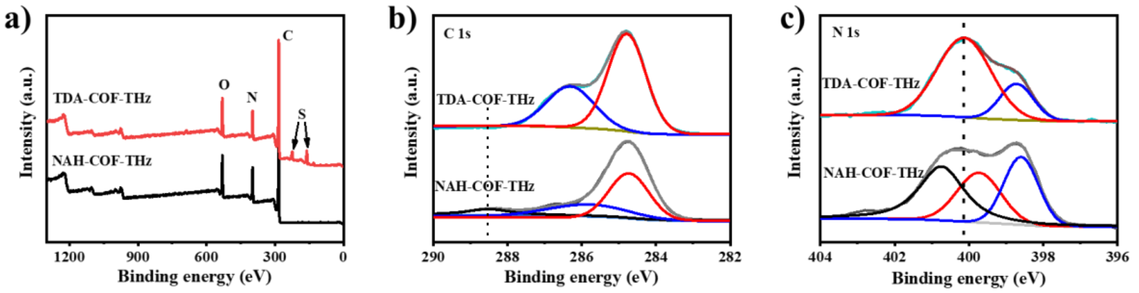

Figure S2. XPS survey spectra, b) C 1s XPS spectra, and c) N 1s XPS spectra of NAH-COF-THz and TDA-COF-THz.

X-ray photoelectron spectroscopy (XPS) survey spectrum of NAH-COF-THz show three different signal, corresponding to C, N and O elements, while two extra signal of S element are discerned for TDA-COF-THz (Figure S2a). As for high-resolution C 1s XPS spectrum of NAH-COF-THz, the peak located at 288.5 eV corresponds to the carbon atom of C=O (Figure S2b). And it was obvious that C=O peak disappeared in TDA-COF-THz, which are in agreement with the FTIR spectrum. The N 1s XPS spectrum of NAH-COF-THz can be resolved into three peak at 398.6, 399.7 and 400.8 eV, which can be assigned to triazine, imine and amide N atom, respectively (Figure S2c). However, there is only two types of N atom for TDA-COF-THz, in which the peak (400.2 eV) corresponds to thiadiazole N atom.

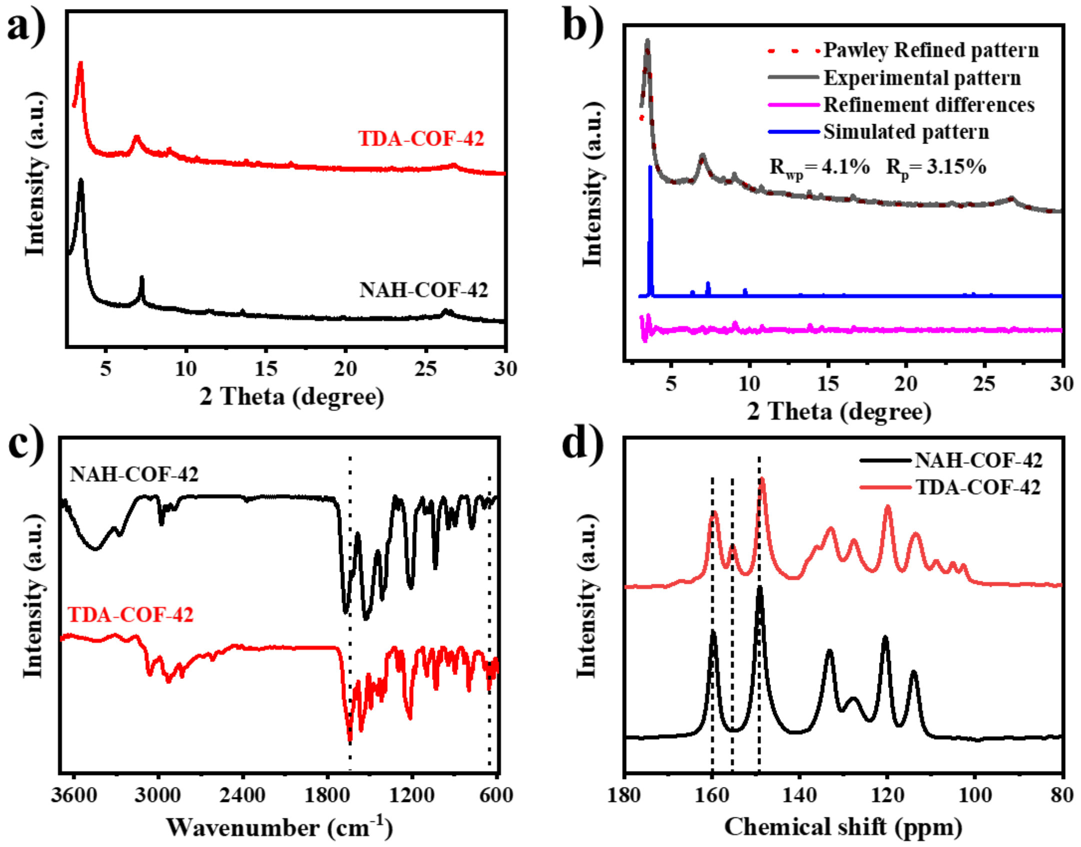

Figure S3. Characterization of NAH-COF-42 and TDA-COF-42: a). XRD patterns. b) Experimental and simulated powder X-ray diffraction patterns of TDA-COF-42. c) FTIR spectra. d) Solid-state ^{13}C NMR spectra.

TDA-COF-42 can maintains good crystallinity after the modification of NAH-COF-42 (Figure S3a). Furthermore, the Pawley refinements of TDA-COF-42 give the unit cell parameters of a =27.87, b = 27.87, and c = 3.79 Å, Rwp = 4.1% and Rp = 3.15% (Figure S3b and Table S3). In the Fourier-transform infrared (FTIR) spectra (Figure S3c), two new peak appeared at 1643 and 653 cm^{-1}, which could be ascribed to the stretching vibration of C=N and C-S in thiadiazole linkage, respectively. Correspondingly, disappearance of C=O and C=N at 1667 and 1620 cm^{-1} in *N*-acylhydrazone motif. In the solid-state ^{13}C NMR spectrum, C=O peak at 159.8 ppm and C=N peak at 149.1 ppm disappear for NAH-COF-42, while TDA-COF-42 presented two new peaks at 159.3 and 155.3 ppm, which were ascribed to the thiadiazole carbon (Figure S3d).

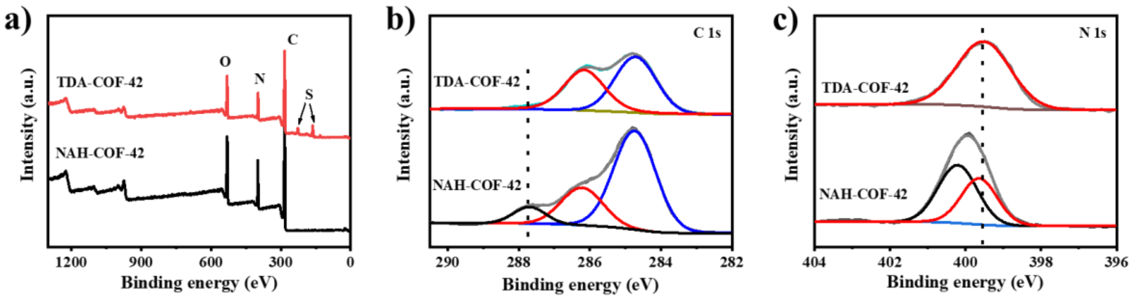

Figure S4. a) XPS survey spectra, b) C 1s XPS spectra, and c) N 1s XPS spectra of NAH-COF-42 and TDA-COF-42.

X-ray photoelectron spectroscopy (XPS) survey spectrum of NAH-COF-42 show three different signal, corresponding to C, N and O elements, while two extra signal of S element are discerned for TDA-COF-42 (Figure S4a). As for high-resolution C 1s XPS spectrum of NAH-COF-THz, the peak located at 287.7 eV corresponds to the carbon atom of C=O (Figure S4b). And it was obvious that C=O peak disappeared in TDA-COF-THz, which are in agreement with the FTIR spectrum. The N 1s XPS spectrum of NAH-COF-THz can be resolved into three peak at 399.7 and 400.2 eV, which can be assigned to imine and amide N atom, respectively (Figure S4c). However, there is only two types of N atom for TDA-COF-THz, in which the peak (399.5 eV) corresponds to thiadiazole N atom.

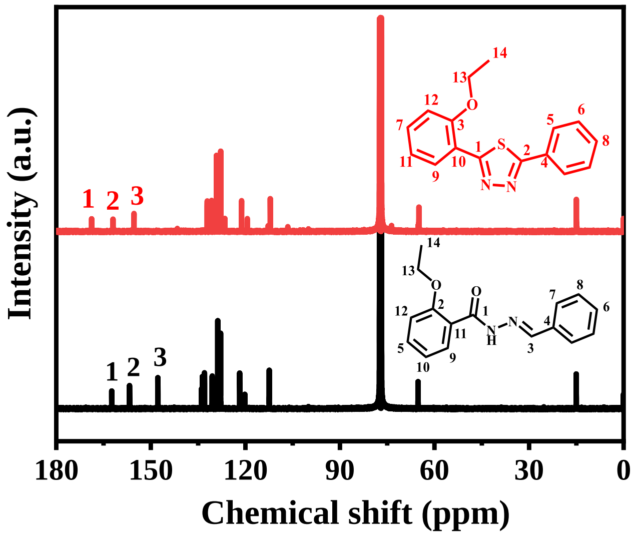

Figure S5. ^{13}C NMR spectra for a) N-acylhydrazone-based and b) thiadiazole-based model compounds.

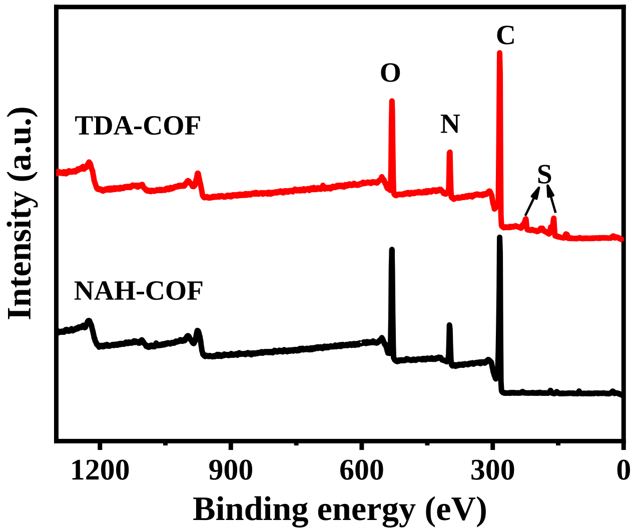

Figure S6. XPS survey spectra of NAH-COF and TDA-COF.

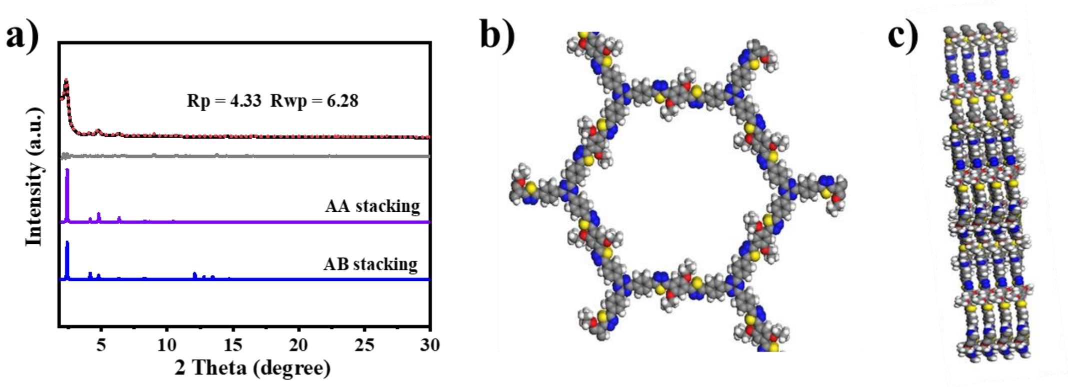

Figure S7. a) The indexed experimental (black), Pawley-refined (red), XRD patterns with their difference (gray) and the theoretical patterns of AA stacking (purple) and AB stacking (blue) of TDA-COF. Top b) and side views c) for AA stacking model of TDA-COF.

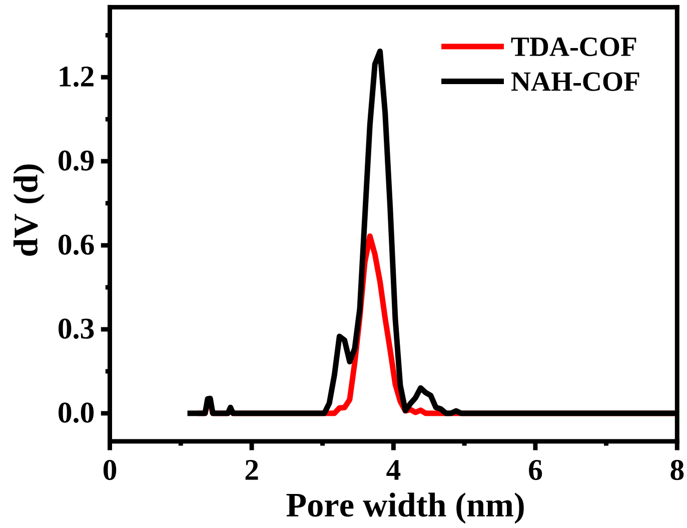

Figure S8. Pore size distributions for NAH-COF and TDA-COF.

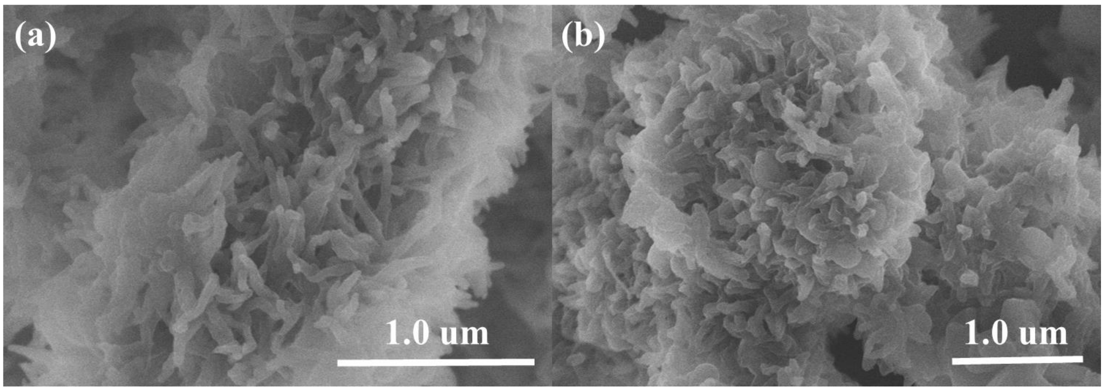

Figure S9. SEM images of a) NAH-COF and b) TDA-COF.

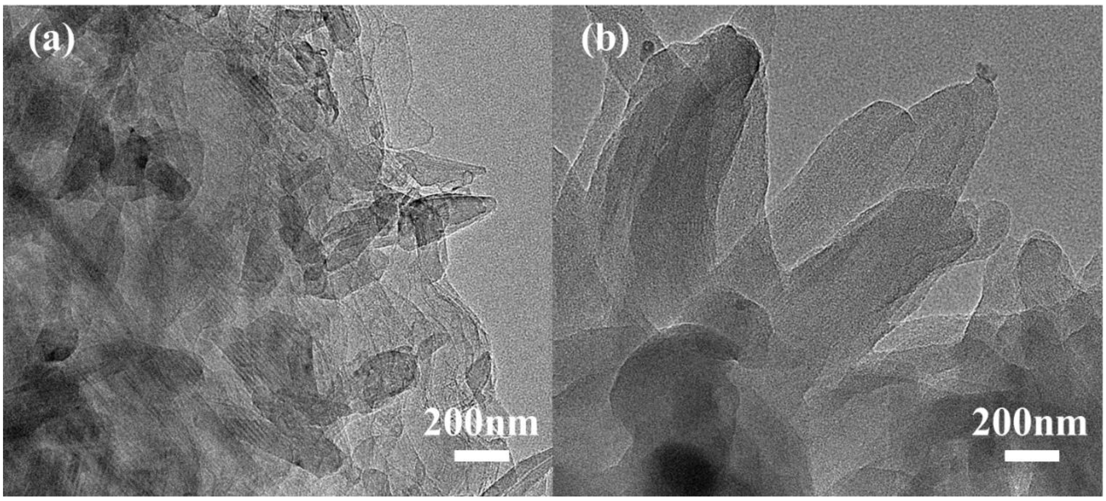

Figure S10. TEM images for (a) NAH-COF and (b) TDA-COF.

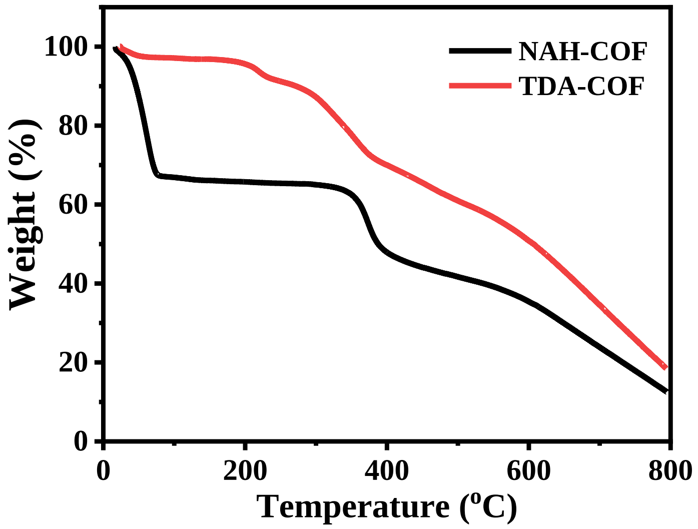

Figure S11. TGA curves of NAH-COF and TDA-COF.

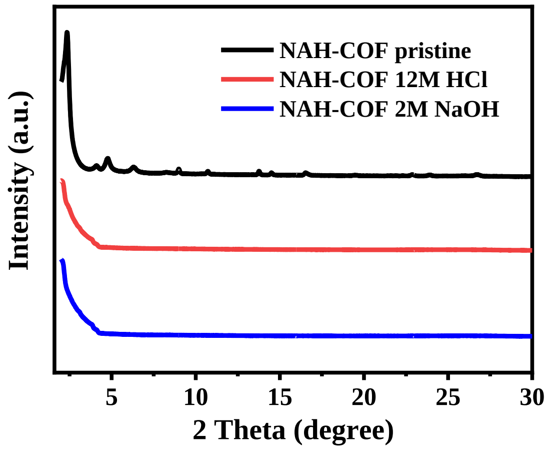

Figure S12. Chemical stability of NAH-COF after treatment under various conditions for 24 h.

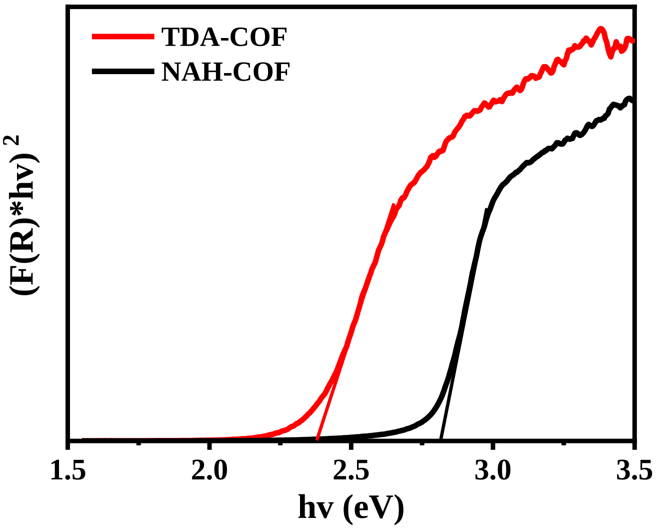

Figure S13. Tauc plots of NAH-COF and TDA-COF.

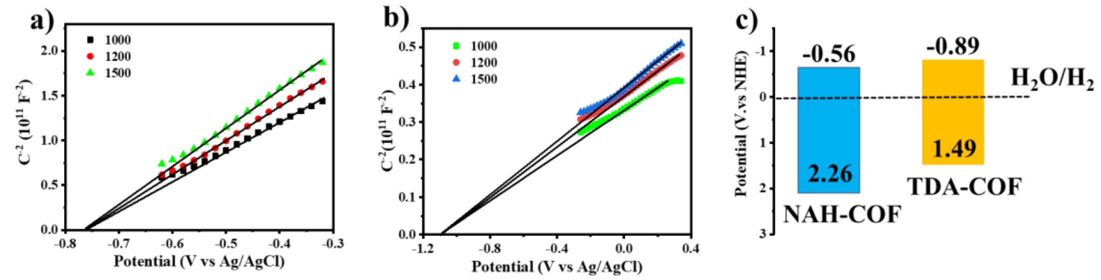

Figure S14. Mott-Schottky plots for a) NAH-COF and b) TDA-COF; c) Band structure diagram of NAH-COF and TDA-COF.

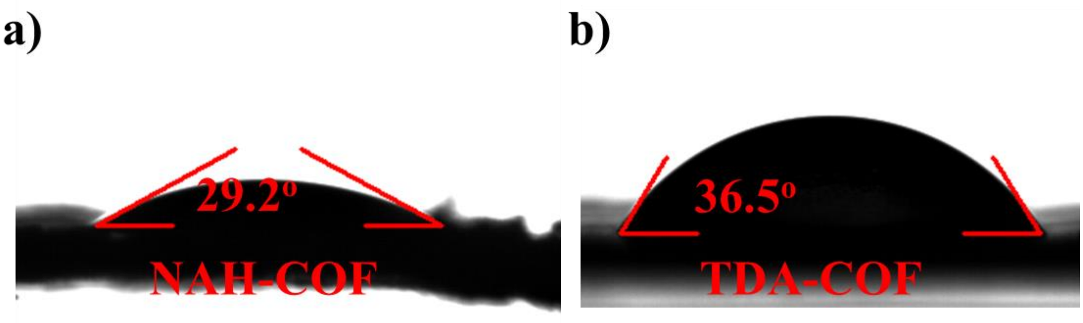

Figure S15. Water contact angles for pressed pellets of a) NAH-COF and b) TDA-COF.

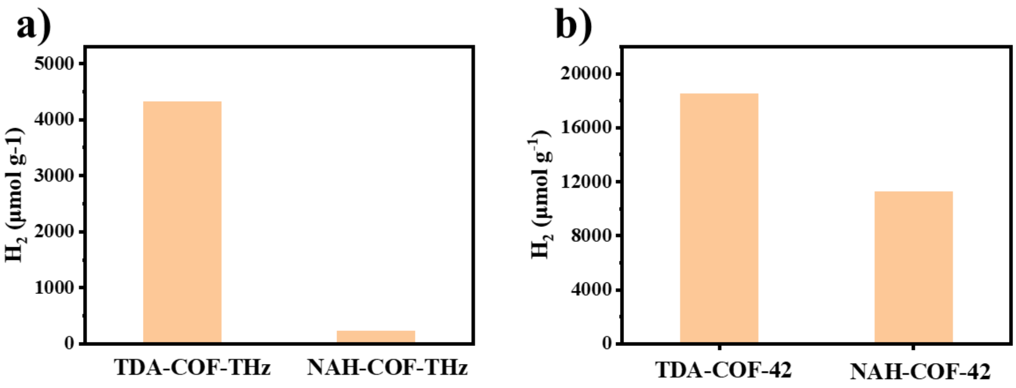

Figure S16. Performance evaluation for Photocatalytic H_2 evolution: a) TDA-COF-THz and NAH-COF-THz, b) TDA-COF-42 and NAH-COF-42.

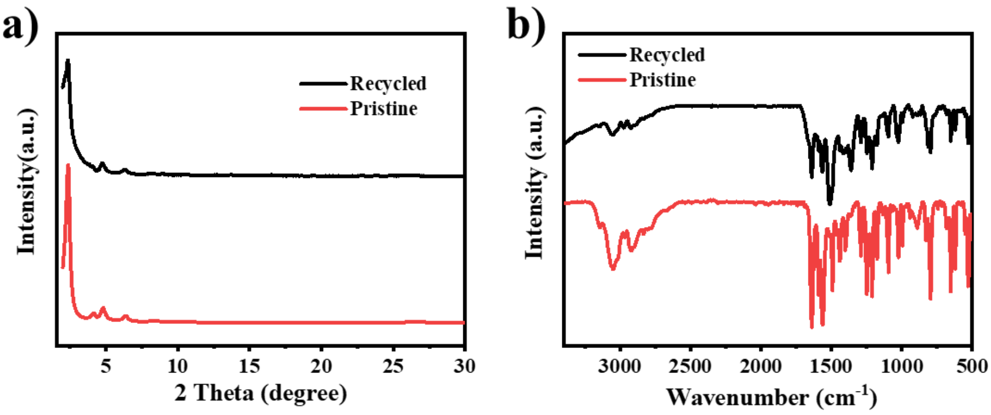

Figure S17. a) XRD patterns and b) FTIR spectra of TDA-COF and TDA-COF after photocatalytic reaction recycling tests.

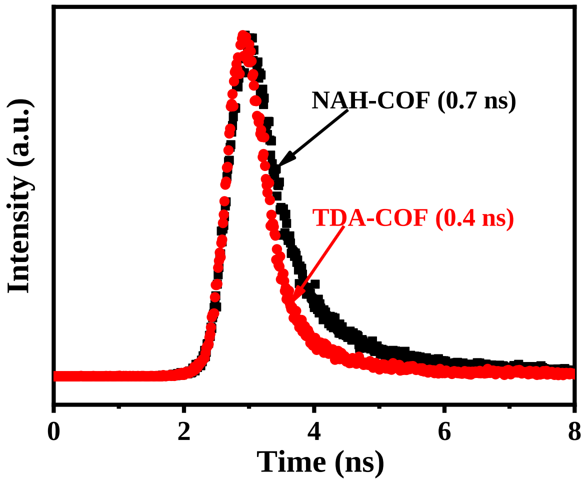

Figure S18. Time-resolved PL decay spectra of NAH-COF and TDA-COF.

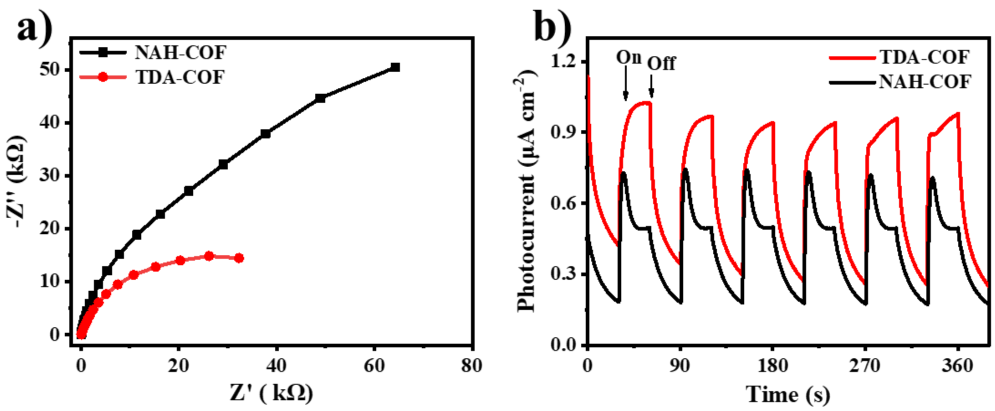

Figure S19. a) EIS Nyquist plots b) Transient photocurrents under visible light irradiation.

Table S1. Atomic coordinates for TDA-COF with AA stacking.

C1	0.48706	-0.47527	5.8E-4
C2	0.46438	-0.51102	-0.12142
C3	0.4777	-0.53542	-0.11791
C4	0.52513	-0.55185	-0.00595
S5	0.56884	-0.54165	-0.08664
C6	0.55338	-0.58715	-0.0468
N7	0.5176	-0.6072	0.01867
N8	0.50175	-0.58744	0.04008
O9	0.42953	-0.52062	-0.25003
H10	0.46099	-0.56293	-0.21123
C11	0.56197	-0.64117	-0.03762
C12	0.58398	-0.6568	-0.05743
C13	0.62148	-0.63514	-0.11534
C14	0.63635	-0.59756	-0.1577
C15	0.61431	-0.58195	-0.13919
C16	0.57687	-0.60354	-0.07653
C17	0.645	-0.65156	-0.12478
N18	-0.31834	-0.63017	-0.12447
H19	0.53325	0.34138	0.01097

H20	0.57162	0.31416	-0.02259
H21	0.6651	-0.5801	-0.20473
H22	0.62682	-0.5529	-0.17209
C23	0.03719	0.59356	0.61511
C24	0.06933	0.62869	0.48222
H25	0.5801	0.56196	1.60853
H26	0.60015	0.57704	1.17426
H27	0.6444	0.55732	1.29184
H28	0.62263	0.53711	1.70829
H29	0.64544	0.58586	1.65283

Table S2. Atomic coordinates for TDA-COF-THz with AA stacking.

C1	0.29837	0.69930	0.00000
C2	0.25781	0.62775	0.00000
C3	0.17915	0.59298	0.00000
C4	0.47398	0.60787	0.00000
C5	0.50500	0.56536	0.00000
C6	0.46351	0.49147	0.00000
C7	0.38964	0.46089	0.00000
C8	0.35677	0.50174	0.00000
C9	0.68546	0.40246	0.00000
C10	0.61218	0.35618	0.00000
C11	0.56182	0.38358	0.00000
N12	0.64859	0.15106	0.00000
N13	0.61479	0.08151	0.00000
C14	0.54396	0.05073	0.00000

S15	0.51374	0.10976	0.00000
H16	0.50726	0.66447	0.00000
H17	0.56175	0.59064	0.00000
H18	0.35641	0.40427	0.00000
H19	0.29990	0.47510	0.00000
H20	0.70547	0.45857	0.00000

Table S3. Atomic coordinates for TDA-COF-42 with AA stacking.

O1	0.46799	0.07191	-0.57754	C61	0.45312	0.57096	-0.10183
C2	0.40481	0.09685	-0.83863	S62	0.38565	0.55345	-0.03194
C3	0.59528	0.95125	0.09787	C63	0.46792	0.94230	-0.29126
C4	0.61136	0.29703	-0.13223	C64	0.52301	0.96527	-0.17095
C5	0.64782	0.27601	-0.15121	C65	0.55834	0.02332	-0.18686
C6	0.59184	0.37450	-0.03836	O66	0.38658	0.45331	-0.25921
N7	0.80387	0.45826	0.18158	C67	0.29676	0.40545	0.02078
N8	0.85793	0.48513	0.23023	C68	0.63866	0.59284	0.33113
C9	0.88133	0.45443	0.14706	C69	0.31159	0.61025	-0.03720
S10	0.83502	0.38916	-0.00437	C70	0.36810	0.64437	-0.11372
C11	0.52045	0.46161	-0.02231	C71	0.21629	0.59496	0.05608
C12	0.55262	0.51797	0.07655	N72	0.34443	0.80799	-0.18739
C13	0.52927	0.55266	0.04674	N73	0.37316	0.86254	-0.21658
O14	0.93028	0.38511	0.23934	C74	0.42759	0.88185	-0.26612
C15	0.90485	0.29714	0.50626	S75	0.44489	0.83084	-0.28858
C16	0.04876	0.64918	0.38081	C76	0.05883	0.52496	0.17267
C17	0.70465	0.31219	-0.09414	C77	0.03531	0.55956	0.19047

C18	0.72501	0.36852	-0.01447	C78	0.97723	0.53525	0.17548
C19	0.62665	0.21630	-0.22279	H79	0.43724	0.12166	-1.03560
N20	0.53700	0.34059	0.00188	H80	0.40803	0.12461	-0.61886
N21	0.50960	0.36784	0.01326	H81	0.36338	0.07974	-0.96292
C22	0.54293	0.42313	-0.01693	H82	0.60359	0.98131	0.31312
S23	0.61158	0.44338	-0.06855	H83	0.62571	0.97243	-0.11670
C24	0.53969	0.05849	-0.31409	H84	0.56756	0.26984	-0.17926
C25	0.48493	0.03556	-0.43938	H85	0.55333	0.59619	0.11394
C26	0.44964	0.97743	-0.42544	H86	0.92137	0.27351	0.64735
O27	0.60675	0.53715	0.20493	H87	0.88112	0.27289	0.27305
C28	0.69374	0.60189	0.47015	H88	0.87645	0.30295	0.68348
C29	0.34604	0.40115	-0.11077	H89	0.02369	0.62763	0.61667
C30	0.68779	0.38860	0.00172	H90	0.02242	0.65566	0.18945
C31	0.63097	0.35323	-0.05768	H91	0.73279	0.29604	-0.10485
C32	0.78465	0.40643	0.05846	H92	0.40746	0.95871	-0.51894
N33	0.66055	0.19586	-0.28569	H93	0.71904	0.59977	0.25164
N34	0.63340	0.14152	-0.32912	H94	0.68645	0.56923	0.66543
C35	0.57818	0.11936	-0.30245	H95	0.71701	0.64332	0.59724
S36	0.55781	0.16691	-0.21510	H96	0.36267	0.38803	0.11236
C37	0.94224	0.47734	0.17087	H97	0.33152	0.36838	-0.31536
C38	0.96574	0.44280	0.19711	H98	0.70310	0.43183	0.06487
C39	0.02382	0.46707	0.18390	H99	0.04212	0.44078	0.17936
O40	0.53989	0.92918	-0.02798	H100	0.64362	0.92081	0.36433
C41	0.60240	0.90393	0.23738	H101	0.59927	0.87596	0.01913
C42	0.41256	0.04970	-0.70337	H102	0.56952	0.87939	0.43284

C43	0.38860	0.70103	-0.17979	H103	0.38215	0.02799	-0.48951
C44	0.35360	0.72384	-0.16724	H104	0.40451	0.02004	-0.92072
C45	0.40603	0.62143	-0.12027	H105	0.43218	0.72744	-0.23679
N46	0.19689	0.54325	0.18168	H106	0.44090	0.39787	-0.21122
N47	0.14291	0.51683	0.23346	H107	0.12000	0.72959	0.25879
C48	0.11974	0.54773	0.14980	H108	0.12483	0.69960	0.66913
S49	0.16625	0.61274	-0.00483	H109	0.07998	0.72913	0.63392
C50	0.47444	0.53187	-0.06638	H110	0.97756	0.37502	0.62877
C51	0.44144	0.47481	-0.14200	H111	0.97859	0.34687	0.20146
C52	0.46533	0.44079	-0.12863	H112	0.26993	0.70613	-0.08104
O53	0.07069	0.61736	0.22576	H113	0.60040	0.04204	-0.09092
C54	0.09638	0.70541	0.49250	H114	0.26600	0.36566	0.14507
C55	0.95236	0.35338	0.39356	H115	0.27657	0.41458	-0.20055
C56	0.29707	0.68881	-0.09335	H116	0.31070	0.43925	0.21785
C57	0.27570	0.63195	-0.02723	H117	0.61626	0.60014	0.54659
C58	0.37620	0.78437	-0.21618	H118	0.64701	0.62315	0.11682
N59	0.46034	0.65308	-0.19275	H119	0.29519	0.56650	0.01406
N60	0.48675	0.62484	-0.18142	H120	0.95894	0.56153	0.16433

Table S4. The activity comparison for TDA-COF and COFs-based photocatalytic systems.

**Catalyst**	**Sacrificial agent**	**pH**	**Activity (mmol g^{-1} h^{-1})**	**Light source**	**Ref.**
TDA-COF	TEOA	Unadjusted	12.26	>420 nm	This work
TtaTfa	AC	Unadjusted	20.7	>420 nm	[S5]
Py-CITP-BT	AC	Unadjusted	8.875	>420 nm	[S6]
PyTA-BC	AC	Unadjusted	5.03	>420 nm	[S7]
PyTz-COF	AC	Unadjusted	2.07	AM 1.5	[S8]
N3-COF	AC	PBS pH 7	0.075	>420 nm	[S9]
A-TEBPY	TEOA	Unadjusted	0.098	AM 1.5	[S10]
PTP-COF	TEOA	PBS pH 7	0.083	AM 1.5	[S11]
TFPT-COF	Sodium ascorbate	Unadjusted	0.23	>420 nm	[S3]
TTR-COF	TEOA	Unadjusted	1.72	>420 nm	[S12]
FS-COF+WS5F	AC	Unadjusted	16.3	>420 nm	[S13]
NTU-BDA-THTA	Sodium ascorbate	PBS pH 7	1.127	>420 nm	[S14]
TpPa-1-COF	Sodium ascorbate	PBS pH 7	1.22	>420 nm	[S15]
TpDTz	TEOA	pH 8.5	0.941	AM 1.5	[S16]
NUS-55	TEA	Unadjusted	0.433	>420 nm	[S17]
Diacetylene-	TEOA	Unadjusted	0.324	>395 nm	[S18]

COF					
ODA-COF	TEOA	Unadjusted	2.615	>420 nm	[S19]
TTAN-COF	TEOA	pH 10.3	11.94	>420 nm	[S20]
COF-932	ascorbic acid	Unadjusted	0.11	AM 1.5	[S21]
COF-JLU100	TEOA	Unadjusted	107.4	>420 nm	[S22]
BTT-BPy-PCOF	AC	Unadjusted	15.8	>420 nm	[S23]
v-2D-COF-NS1	TEOA	Unadjusted	4.4	>400 nm	[S24]
EnTAPT-TDOEB	ascorbic acid	PBS pH 7	2.396	≥ 380 nm	[S25]
g-$C_{40}N_3$-COF	TEOA	Unadjusted	4.12	>420 nm	[S26]

References.

[S1] X. Li, Q. Gao, J. Wang, Y. Chen, Z. H. Chen, H. S. Xu, W. Tang, K. Leng, G. H. Ning, J. Wu, Q. H. Xu, S. Y. Quek, Y. Lu, K. P. Loh, *Nat. Commun.*, **2018**, *9*, 2335.

[S2] A. Cutler, I. Ko, S. Park, G. Lee, H. Kim, *Arkivoc*, **2019**, *2019*, 67.

[S3] L. Stegbauer, K. Schwinghammer, B. V. Lotsch, *Chem. Sci.*, **2014**, *5*, 2789.

[S4] F. J. Uribe-Romo, C. J. Doonan, H. Furukawa, K. Oisaki, O. M. Yaghi, *J. Am. Chem. Soc.*, **2011**, *133*, 11478.

[S5] J. Yang, A. Acharjya, M.Y. Ye, J. Rabeah, S. Li, Z. Kochovski, S. Youk, J. Roeser, J. Gruneberg, C. Penschke, M. Schwarze, T. Wang, Y. Lu, R. van de Krol, M. Oschatz, R. Schomacker, P. Saalfrank, A. Thomas, *Angew. Chem. Int. Ed.*, **2021**, *60*, 19797.

[S6] W. B. Chen, L. Wang, D. Z. Mo, F. He, Z. L. Wen, X. J. Wu, H. X. Xu, L. Chen, *Angew. Chem. Int. Ed.*, **2020**, *59*, 16902.

[S7] A. F. M. El-Mahdy, A. M. Elewa, S. W. Huang, H. H. Chou, S. W. Kuo, *Adv. Opt. Mater.,* **2020**, *8,* 2000641.

[S8] W. Li, X. Huang, T. Zeng, Y. A. Liu, W. Hu, H. Yang, Y. B. Zhang, K. Wen, *Angew. Chem. Int. Ed.,* **2021**, *60,* 1869.

[S9] V. S. Vyas, F. Haase, L. Stegbauer, G. Savasci, F. Podjaski, C. Ochsenfeld, B. V. Lotsch, *Nat. Commun.,* **2015**, *6,* 8508.

[S10] L. Stegbauer, S. Zech, G. Savasci, T. Banerjee, F. Podjaski, K. Schwinghammer, C. Ochsenfeld, B. V. Lotsch, *Adv. Energy Mater.,* **2018**, *8*, 1703278.

[S11] F. Haase, T. Banerjee, G. Savasci, C. Ochsenfeld, B. V. Lotsch, *Faraday Discuss.,* **2017**, *201*, 247.

[S12] L. Y. Li, Z. M. Zhou, L. Y. Li, Z. Y. Zhuang, J. H. Bi, J. H. Chen, Y. Yu, J. G. Yu, *Acs Sustain. Chem. Eng.,* **2019**, *7,* 18574.

[S13] X. Y. Wang, L. J. Chen, S. Y. Chong, M. A. Little, Y. Z. Wu, W. H. Zhu, R. Clowes, Y. Yan, M. A. Zwijnenburg, R. S. Sprick, A. I. Cooper, *Nat. Chem.,* **2018**, *10*, 1180.

[S14] H. Wang, C. Qian, J. Liu, Y. F. Zeng, D. D. Wang, W. Q. Zhou, L. Gu, H. W. Wu, G. F. Liu, Y. L. Zhao, *J. Am. Chem. Soc.,* **2020**, *142*, 4862.

[S15] F. M. Zhang, J. L. Sheng, Z. D. Yang, X. J. Sun, H. L. Tang, M. Lu, H. Dong, F. C. Shen, J. Liu, Y. Q. Lan, *Angew. Chem. Int. Ed.,* **2018**, *57*, 12106.

[S16] B. P. Biswal, H. A. Vignolo-Gonzalez, T. Banerjee, L. Grunenberg, G. Savasci, K. Gottschling, J. Nuss, C. Ochsenfeld, B. V. Lotsc, *J. Am. Chem. Soc.,* **2019**, *141*, 11082.

[S17] J. Wang, J. Zhang, S. B. Peh, G. L. Liu, T. Kundu, J. Q. Dong, Y. P. Ying, Y. H. Qian, D. Zhao, *Sci. China Chem.,* **2020**, *63*, 192.

[S18] P. Pachfule, A. Acharjya, J. Roeser, T. Langenhahn, M. Schwarze, R. Schomacker, A. Thomas, J. Schmidt, *J. Am. Chem. Soc.,* **2018**, *140*, 1423.

[S19] S. Yang, H. Lv, H. Zhong, D. Yuan, X. Wang, R. Wang, *Angew. Chem. Int. Ed.,* **2022**, *61*, e202115655.

[S20] Y. Yang, N. Luo, S. Lin, H. Yao, Y. Cai, *ACS Catal.*, **2022**, *12*, 10718.

[S21] W. Dong, Z. Qin, K. Wang, Y. Xiao, X. Liu, S. Ren, L. Li, *Angew. Chem. Int. Ed.*, **2023**, *62*, e202216073.

[S22] S. Ma, T. Deng, Z. Li, Z. Zhang, J. Jia, G. Wu, H. Xia, S. W. Yang, X. Liu, *Angew. Chem. Int. Ed.*, **2022**, *61*, e202208919.

[S23] L. Dai, A. Dong, X. Meng, H. Liu, Y. Li, P. Li, B. Wang, *Angew. Chem. Int. Ed.*, **2023**, e202300224.

[S14] S. Li, R. Ma, S. Xu, T. Zheng, H. Wang, G. Fu, H. Yang, Y. Hou, Z. Liao, B. Wu, X. Feng, L.-Z. Wu, X.-B. Li, T. Zhang, *ACS Catal.*, **2023**, *13*, 1089.

[S25] X. Guan, Y. Qian, X. Zhang, H. L. Jiang, *Angew. Chem. Int. Ed.*, **2023**, e202306135.

[S26] S. Bi, C. Yang, W. Zhang, J. Xu, L. Liu, D. Wu, X. Wang, Y. Han, Q. Liang, F. Zhang, *Nat. Commun.*, **2019**, *10*, 2467.
